# Adult mental health clinicians’ perspectives of parents with a mental illness and their children: single and dual focus approaches

**DOI:** 10.1186/s12913-018-3428-8

**Published:** 2018-08-06

**Authors:** Phillip Tchernegovski, Rochelle Hine, Andrea E. Reupert, Darryl J. Maybery

**Affiliations:** 1 Krongold Clinic, Faculty of Education, Monash University, Clayton Campus, Melbourne, VIC 3800 Australia; 2 South West Healthcare, Ryot Street, Warrnambool, VIC 3280 Australia; 3 Department of Rural and Indigenous Health, School of Rural Health, Monash University, PO Box 973, Moe, VIC 3825 Australia

**Keywords:** Parental mental illness, Workforce issues, Family-focused practice, Interpretative phenomenological analysis

## Abstract

**Background:**

When clinicians in the adult mental health sector work with clients who are parents with dependent children, it is critical they are able to acknowledge and respond to the needs of the parents and their children. However, little is known about clinicians’ personal perspectives and reactions towards these parents and children or if/how they balance the needs of both.

**Methods:**

Semi structured interviews were conducted with eleven clinicians from adult mental health services in Australia. Interviews focused on clinicians’ experiences when working with parents who have mental illness. Transcripts were analysed within an Interpretative Phenomenological Analysis framework to examine participants’ perspectives and personal reactions to parents and children.

**Results:**

There was considerable divergence in participants’ reactions towards parents and children and the focus of their perspectives when working with parental mental illness. Feelings of sympathy and responsibility made it difficult for some participants to maintain a dual focus on parents and children and contributed to some adopting practices that focused on the needs of parents (*n* = 3) or children (*n* = 1) exclusively. Other participants (*n* = 7) described strategies and supports that allowed them to manage these feelings and sustain a dual focus that incorporated the experiences and needs of both parents and children.

**Conclusions:**

It is difficult for some mental health clinicians to maintain a dual focus that incorporates the needs and experiences of parents and their children. However, findings suggest that the challenges of a dual focus may be mitigated through adequate workplace support and a strengths-based practice framework that emphasises parental empowerment.

## Background

Up to one third of adults who access mental health services are parents caring for dependent children [1]. Managing a mental illness may make it difficult for them to meet the demands of parenting [2, 3] and may disrupt parenting behaviours and the parent-child relationship in a variety of ways. For example, some parents may become inattentive [4], hostile and aggressive [5] or controlling [6]. In response to the diminished capacity of some parents, children may assume responsibilities, such as caring for siblings or the parent [4, 7]. These dynamics, as well as genetic and other environmental factors, may lead children to develop substance abuse problems [8], behavioural disorders [9] or their own mental illness [10, 11]. Parents may experience shame and guilt about their parenting difficulties and the impacts of their illness on children, which can in turn, exacerbate their mental illness [12]. To effectively support these parents and their children, mental health clinicians must understand and respond to issues pertaining to parenting with a mental illness [13]. Hence, a whole-family perspective is required.

Internationally, several initiatives have emerged related to mental illness, parenting and child wellbeing. Policy and legislation has been introduced in some countries that require adult mental health services to identify the children of people who are receiving services and provide them with appropriate information and/or support [14, 15]. Victoria is the first Australian state to legislate on this issue. Section J of the Mental Health Act (2014) [15] mandates that ‘children, young persons and dependents of persons receiving mental health services should have their needs, wellbeing and safety recognized and protected.’ However, the remainder of the Act lacks clear directives about how this is to be achieved. Practice guidelines for addressing parental mental illness have also been developed in Australia [16]. While these practice guidelines are not compulsory, they outline specific areas of best practice such as the recognition and support of family needs, provision of information to family members and actions to ensure the care and protection of children.

Various programs have been developed for supporting families when a parent has a mental illness. These include programs for children [17] and whole family interventions [18]. Structured, manualised interventions have also been developed that may be delivered to the parent with the mental illness by clinicians working in adult mental health [19, 20]. One such intervention is Let’s Talk about Children [21] which involves the clinician and the parent discussing ways that the parent might strengthen family relationships and promote the healthy development of their child. Overall, family-focused interventions are effective for promoting positive outcomes for the parent experiencing a mental illness, their children and the family unit [22, 23]. Accordingly, the adult mental health sector is an ideally positioned to support families where parents have a mental illness.

While initiatives such as policy and development of interventions are vital, it is also important to understand the experiences and perspectives of clinicians who work with parents. Most research in this area has focused on organisational culture, which generally does not promote family-focused work [24] and the lack of time and resourcing available to clinicians to undertake family-focused practices [25]. Research has also highlighted that clinicians believe that they lack skill and knowledge for working with parental mental illness [24] and may experience anxiety related to undertaking specific tasks such as raising concerns about child safety with parents [26, 27]. Meanwhile, little is known about clinicians’ beliefs, attitudes and emotional reactions towards parents or their children. Clinicians’ personal reactions may be incongruent to their external behaviours [28]. For example, clinicians may behave empathetically to support an individual’s mental health, despite viewing the person negatively or experiencing feelings of frustration. Scott [29] suggested that clinicians’ personal reactions towards parenting and child wellbeing may be particularly intense. Given the potential complexity and intensity of clinicians’ personal reactions towards parents and children further research in this area is needed to inform initiatives to support clinicians when working with parental mental illness.

Literature relating to clinicians’ personal reactions towards parents and their children is sparse. Early research focused on countertransference processes of child welfare clinicians, who reportedly characterised family members as victims and/or perpetrators [30]. Clinicians’ feelings of fear, guilt, shame, anger and sympathy were also considered to be common aspects of this countertransference towards parents and children [31]. Killen [32] suggested that clinicians employed a range of defensive strategies to protect themselves from the emotional burdens of witnessing child abuse and neglect. These strategies included over-identification with parents (which allowed clinicians to avoid witnessing the hardships of the child) and over-simplified treatment approaches (which avoided a full consideration of potentially distressing family situations).

Relatively more recent literature has considered the tensions between the child-centric perspectives of child protection services and the parent-centric perspectives of adult mental health services [33]. Fleck-Henderson [34] argued that it is beneficial for clinicians from both service sectors to “see double” (p. 333) by simultaneously maintaining perspectives of parents *and* children. Nonetheless, Cousins [35] argued that the needs of parents and children are often in conflict, which makes it difficult for clinicians to balance both perspectives, especially when affiliated with a workplace that prioritises parents’ mental health. Although the arguments made by Fleck-Henderson and Cousins are supported by their own practice, there is an absence of empirical research relating to how mental health clinicians direct their attention towards parents and/or their children.

This current paper aims to address important gaps in the literature by examining mental health clinicians’ perspectives and personal reactions to the parents they work with and their children. The research was driven by the following research questions:

- What are clinicians’ personal reactions towards parents and their children?
- How, if at all, do clinicians attempt to maintain a concurrent focus on the needs of parents and their children? If they do, what are their experiences of this?

An understanding of these intra-personal experiences can inform the development of future training, workplace policy and practice and resources to support for clinicians when working with parents.

## Method

### Participants

Participants were recruited from Australian clinical adult mental health services. Although mental health legislation is managed at a state level, services are guided by national standards and have common workplace structures [36]. Thus, clinicians from all states and territories of Australia were eligible for inclusion. A sample of 11 participants was interviewed from Victoria, Western Australia and New South Wales.

The Australian clinical adult mental health sector provides a range of services from triage, assessment and inpatient treatment, through to community-based rehabilitation [37]. The target population for clinician adult mental health services are adults between 16 and 64 years who are experiencing significant mental health disturbance, crisis situations or severe mental illness [38]. Multidisciplinary team structures are utilised in the clinical adult mental health sector. All team members share common tasks and responsibilities while also contributing particular knowledge and expertise that is specific to their professional background and training [39, 40].

The mean age of participants was 39.3 (SD = 10.1). Participants had a mean of 8.4 (SD = 6.2, Range = 2.5–20) years of experience working in the adult mental health sector and 3.9 (SD = 2.8, Range = 0–9) years of experience of working with children. Additional demographics for each participant are presented in Table 1. Pseudonyms are used to protect the privacy of participants.

**Table 1:** Self-reported participant demographics

Pseudonym	Gender	Profession	Work Setting	Geography of workplace
Claudia	Female	Psychologist	Community^c^	Regional
Craig	Male	Psychologist	Inpatient^a^	Rural
Angela	Female	Psychologist	Inpatient^a^	Regional
Emily	Female	Psychologist	Outpatient^b^	Rural
Michael	Male	Social Worker	Outpatient^b^	Suburban
Katherine	Female	Social Worker	Inpatient^a^	Rural
Kelly	Female	Social Worker	Outpatient^b^	Suburban
Kurt	Male	Mental Health Nurse	Community^c^	Suburban
Frances	Female	Mental Health Nurse	Community^c^	Regional
Vicki	Female	Psychiatrist	Outpatient^b^	Suburban
Julie	Female	Occupational Therapist	Community^c^	Regional

^a^Inpatient = parent resides at mental health service while receiving treatment

^b^Outpatient = parent attends mental health service for scheduled appointments

^c^Community = parent is visited at home or in a community setting by the mental health clincian(s)

### Procedure

A recruitment email was disseminated through the researchers’ professional networks, including mental health managers, clinicians and practice development professionals. The email contained an explanatory statement about the research and sought expressions of interest from potential participants. A request was also made for recipients to forward the email through their professional networks. Expressions of interest were received from 11 participants from the mental health sector. Given it is unknown how many professionals were forwarded the recruitment information, it is not possible to calculate a response rate.

Participant consent and demographic information were obtained before semi-structured telephone interviews were completed. Interview length ranged from 18 to 43 min (averaging 27 min). Interviews were audio recorded and transcribed, with participant permission. After personal details were removed from transcripts, they were emailed to participants who were then given the opportunity to add information or remove anything that they believed was identifying. Two participants made minor additions. Ethics approval for this study was provided by the university human research ethics committee.

### Interview

The semi-structured interview schedule was developed by the authors specifically for this study. It consisted of open-ended questions designed to broadly examine clinicians’ experiences when working with parents. These were then followed up with probing questions to obtain more detail. Key questions from the interview schedule and some of the probing questions used in interviews were:

1. What is it like for you to work with parents who have mental illness?
  Example probes: How is that different to working with people who are not parents? Is that typical of your work with most parents? What is your main objective when working with parents?
2. Do you consider the needs of the parent *and* the child? If so, what is that experience like?
  Example probes: Who is your main focus? Do you find the two perspectives compatible? How do you manage that?
3. What emotions, if any, do you experience when working with parents?
  Example probes: When do you feel that way? Does that feeling impact on your work with the parent? How do you manage those feelings?

### Analysis

Interview data was analysed using Interpretative Phenomenological Analysis (IPA) because it is a methodology for examining participants’ experiences and perspectives of phenomena [41]. Thus, IPA is well suited to the objectives of investigating clinicians’ perspectives and personal reactions when working with parents. The first of several analytic steps involved reviewing the interview transcripts multiple times to identify the main points communicated by each participant. Transcripts were then coded, whereby short sections of the transcripts were labelled with key phrases, ideas and contextual information. The codes were revised multiple times and used to identify categories and themes relating to participants’ perceptions of parents with a mental illness and their children. The analysis was primarily conducted by the first author, with a separate analysis of five transcripts undertaken by the second author. Interpretation differences were managed by further reviewing the transcripts. All authors also engaged in reflective conversations about possible interpretations of the interview data and themes throughout the study.

## Results

There was considerable variability in participants’ perspectives of parents and children. Participants could broadly be categorised into one of three groups who focused on the parent, the child or both. Three themes, each with two subthemes, were identified. They are listed in Table 2 and described further below. Representative quotes are tagged with participant pseudonyms from Table 1.

**Table 2:** Themes and subthemes

Themes	Subthemes
-Clinicians’ perspectives of parents and their children	-Seeing difficulties
	-Seeing strengths
-Single focus: Seeing the parent *or* the child	-Focusing on parents
	Focusing on children
-Dual focus: Seeing the parent *and* the child simultaneously	-Experiences of dual focus
	-Maintaining the dual focus

### Clinicians’ perspectives of parents who experience mental illness and their children

Participants’ perspectives varied from sympathetic and hopeless views of parents and their children to more hopeful and optimistic views. Some participants focused on difficulties, including the disempowerment of parents and the vulnerabilities of their children. Meanwhile, others emphasised the strengths and motivations of parents and hope about achieving positive outcomes for children.

#### Seeing difficulties

All participants recognised that parents felt disempowered in many ways. Six participants stated that parents were disempowered by child-focused services who “scrutinised” (Claudia) or restricted their parenting. Some also saw other family members as contributing this disempowerment, through a lack of support and criticism. For example, when describing parents who have a mental illness Frances reported that “they never get to make a choice about anything. Everybody thinks they know better… so they really never learn these parenting skills because they’ve always been told themselves what to do… because they’re sort of at this stunted level in development in everybody else’s eyes.” This disempowerment of parents by services and families was seen to sometimes create a “very hopeless place for [these parents] which is very sad and distressing for them.” (Frances).

In relation to children, seven participants viewed them as vulnerable to the impacts of their parents’ behaviours and to the intergenerational cycle of mental illness. Consequently, five participants reported strong feelings of sympathy, fear and/or responsibility towards the children. For example, Kelly said; “Sometimes I do feel quite sorry for those children because you can see them getting caught up in those patterns of behaviour because they are learning some of that.” Claudia recognised that her emotional responses towards these children had intensified since she became a mother herself; “when I see children that are babies or children the same ages to my child - that, for me, is quite confronting.”

Vicki recognized that such emotions occurred when she identified with the powerlessness of the child’s situation; “I think there is so much fear around mental illness and so much fear that children will be harmed or neglected… I think it relates to the vulnerability of the child… to identify with the vulnerability of the child… can add to those very powerful feelings for the worker.”

#### Seeing strengths

Eight participants acknowledged that being a parent was a meaningful experience; “Peel it all back and there’s a child there at the heart of it” (Michael). These participants believed that most parents were dedicated and hopeful about their parenting, as demonstrated by Emily’s comment that “parents really, really, really want to do a good job for the most part.” Consequently, being a parent was seen as a strong motivating factor to work harder towards mental health recovery; “The main motivation is their children… they do want to give well more than 100%” (Julie).

Six participants were also hopeful for the outcomes of these children. For example, Katherine said that these children held “potential and possibility”. These participants viewed their own work with the parent as “early intervention” (Claudia) and an opportunity to “break that cycle” (Emily) of intergenerational mental illness.

### Single focus: Seeing the parent *or* the child

Three participants with particularly strong sympathetic perspectives of parents had developed a singular focus of those parents, with minimal attention paid to their children. Similarly, five participants became heavily focused on children when triggered by sympathetic feelings towards them. While this child-focus was transient for four of these participants, one participant remained constantly focused on children.

#### Focusing on parents

While all participants acknowledged that the wellbeing of children was paramount, three participants (Angela, Craig and Julie) reportedly attended to child wellbeing only if there were signs of abuse or neglect. They did this by reporting the issue to child protection services. These participants remained focused on the presenting issues of the parent; “I’m much more client focused about ‘What are you here for?’” (Angela). When they discussed parenting issues, it was from the parent’s perspective such as to “talk about the stress of parenting” or “let off steam” (Craig).

These three participants felt especially sympathetically towards parents due to their disempowerment by services and families. This was especially meaningful for Angela and Julie who had supported multiple parents during or after the removal of their children by child protection services: “It effects the parent in a huge way… and that doesn’t seem to be looked into as much as it should be” (Julie).

These participants assumed that other clinicians or services would address the wellbeing of these children. For example, Angela, a psychologist, said; “The social workers do a lot of the family work and the doctors might talk to the family for collateral history and things like discharge planning. I’m a bit more one-on-one therapy with the client themselves.”

#### Focusing on children

Five participants reported becoming overwhelmed by sympathy for children and became disproportionately focused on these needs of children. Further, three of these participants had experienced negative feelings and judgments towards the parents. This was evident when Kurt commented; “If you’ve got one job in life, try and do it correctly - being a parent. You know, you feel like saying ‘Put more effort in!’”

The following comment suggests that Kurt also felt helpless when attempting to support children who he believed “have a high expectation of you.” He commented; “It’s difficult to leave a kid in a crappy situation. You don’t want to and you want things to improve the next day but you’re leaving that kid in a less than ideal - by a long shot - environment.”

While most participants commented that their emotionally charged focus on children was temporary, Kurt prioritised “the child in front of the parent” most of the time.

In order to advocate for these children, Kurt believed that it was necessary to “take on an authoritarian role instead of a clinician role… you’ve moved across the line from someone who’s trying to help them to someone who’s against their will.” He recognised that a limitation of this approach was that parents sometimes thought that he was “judging them” and so became “very defensive.”

### Dual focus: Seeing the parent *and* the child simultaneously

Seven participants attempted to maintain a focus on parents and children simultaneously. However, they found this challenging and sometimes became temporarily focused on party or the other. They had developed strategies for managing the difficulties of maintaining this dual focus.

#### Experiences of dual focus

The concurrent view of parents and children was described by Vicki as “a dual focus… there needs to be a focus on the child outcomes and wellbeing and there needs to be a focus on the parent outcomes and wellbeing.” Within this dual focus, five participants said that their goal was to support the parent with their mental health and parenting so that the parent was able to meet the needs of their children; “If we can make a difference for these parents so that they can do what they need to do to meet their kids … then that’s kind of best for everybody” (Emily).

When utilising a dual focus, participants had experienced conflict between attending to the needs of the parent and the needs of the children. They described this as “a real balancing act” (Vicki), “walking a line” (Katherine), a “tight rope” (Frances), a “push and pull” (Michael) and “balancing back and forth” (Claudia). These tensions were especially intense for participants when faced with formal decisions, such as making child protection repots. This was illustrated in a comment made by Frances; “It gives you a few sleepless nights because you wonder what the impact will be on the parents if the welfare turn up.” Five of these participants also experienced the tensions of a dual focus when adequate support was not available from services, families and the community for the family; “in an ideal world, there would be sufficient support … to support the parent to do what they can do and to make up for the parts that they can’t do … There seems to be never enough” (Vicki).

#### Maintaining the dual focus

Participants had developed several strategies for balancing their perspectives of parents and children, and associated emotions. Four participants remained mindful of their focus and purposefully alternated between perspectives of the parent and the child, as necessary to help manage emotional reactions. For example, Kelly described her deliberate efforts to empathise with a father after becoming frustrated at what she perceived to be his unprotective parenting behaviours; “I had to sort of push through that and keep engaging with him… understanding the consumer’s story a lot better… he did want to act protectively but that his illness made it difficult for him.”

Five participants managed sympathetic feelings towards parents and children by utilising a strengths-based approach to empower parents and families. Kelly highlighted the benefits of this approach “… using the strengths based words and reminding them of the hope that there is … self-care in itself because we’re working towards something better together. And I think working towards those goals step-by-step also helps clinicians to remind themselves that there is hope and that it’s not just an endless cycle.”

Similarly, Frances discussed the benefits of a strengths-based approach to counteract her feelings of sympathy and parents’ feelings of helplessness. She commented, “there’s a lot that’s positive … [even] when you see a family who have been really struggling and thinking that they’re not going anywhere.” By focusing on these positive factors and strengths, she encouraged families, and herself, to realise that “… we might not be perfect but then nobody is ... It’s not all bad and we are not all bad as parents. We can still be a family. We can still love our children, nurture them and give them the best that we can which is the same as any other parent would do.”

Participants also promoted parental empowerment by encouraging parents to make their own decisions about their mental health and parenting. Katherine commented; “if I decide to do anything, then immediately I’m creating a sort of semi-resistance to this woman’s capacity to do it for herself.” Likewise, Michael clarified that his role with parents “… is not telling them what to do, but to allow them to be well informed and give them a range of options.”

Those participants who attempted to maintain a dual focus benefited from support within their workplace. This support came in multiple formats, including “supervision” (Kelly), debriefing (Frances), “team consultation, team supervision, team discussion” (Emily), formal and informal “multidisciplinary team” discussions (Michael), clinical review (Claudia), and consultations with specialists such as “FaPMI” (Kurt) co-ordinators who specialise in families where a parent has a mental illness.

Four participants appreciated that their colleagues were less emotionally involved in their cases than themselves, so could offer objectivity and a redirection towards a balanced perspective of parents and children when necessary. Support from these co-workers helped clinicians decide “… which direction we go in and the timing of it. And I find that clinical support is very helpful in knowing where to go next” (Vicki).

In addition to support for decision making when working with parents, clinicians also valued the emotional support that they received from within their organisations. Emily said this support allowed her to “accept that you’re a human and you’re going to have emotional reactions to things as well and that’s okay. Let’s just have a quick de-brief and then you’ll be okay.”

## Discussion

This study examined the self-reported perceptions and personal reactions of mental health clinicians within the Australian adult mental health sector towards parents who have a mental illness and their children. Overall, participants’ perspectives of parents and children were emotionally laden which influenced the particular focus on their work in terms of who they supported. Clinicians in this study tended to approach their work with parents in one of three ways – focusing on the parent, the child or both.

Three participants focused solely on the needs of parents. Although they acknowledged mandates to report suspected incidents of abuse or neglect, they did not actively enquire about child wellbeing. Early research by Killens [32] suggested that clinicians may choose to focus solely on parents so that they do not have to witness the hardships of children. In contrast, the parent-only focus of some participants in this study may have been driven by a sense of loyalty towards the parents. These participants perceived parents as being distressed and disempowered by child-focused services and family members. Thus, they viewed the advocacy of children as conflicting with the support of the parents. This is consistent with Cousins’ [35] assertion that clinicians within adult mental health services may feel compelled to side with parents. By maintaining a focus on the parent, and not the child, participants may have affirmed their alliance with the parent, thereby reducing their own internal tensions and tensions in their interactions with parents.

Organisational factors may also contribute to clinicians adopting a singular parent-focus. A lack of an organisational policy to promote and guide the support of children has been well documented [13, 24]. Although recent legislation in the state of Victoria mandated the support of children whose parents receive mental health services, one study found that the support of children was not consistently implemented as a priority nor enforced within organisations as had been achieved with sections of the Act that had been supported with clearer directives and resourcing [42]. Thus, it is perhaps understandable that participants from this study and previous research [30, 31, 43, 44] claimed that issues related to parenting and child wellbeing were the responsibilities of other professionals rather than within their own remit. In the absence of strongly endorsed policy within organisations, the perception that the wellbeing of the children of parents with mental illness is beyond the clinician’s role is easily promulgated.

It may be common for clinicians in the adult mental health sector to focus exclusively on parents, with many clinicians failing to identify the presence of dependent children or consider them in case planning [45, 46]. Clinicians may assume that issues related to parenting and child wellbeing will be adequately addressed by supporting the parent’s mental health [47]. However, reductions in parents’ mental health do not always led to beneficial outcomes for children [48, 49]. For example, even with a reduction in a parent’s mental health symptoms, disruptions to the parent-child relationship may persist and negatively impact on child wellbeing [50]. Thus, the needs of children are unlikely to be reliably identified and supported by clincians who have a singular focus on parents.

In contrast, other participants reported times that they became focused on risks posed to children by the parents’ mental illness and associated parenting behaviours. This child-focus occurred when they experienced feelings of sympathy, responsibility and a sense of hopelessness for these children. While this focus on children was constant and pervasive for one participant, others reported it as being a temporary stance. Gladstone, Boydell and McKeever [51] suggested that the risks to children are often over-emphasised which may result in them being characterised as passive victims. They argue that this perspective of children does not acknowledge their capabilities nor allow them to actively contribute to the planning of their parents’ recovery or the promotion of their own wellbeing. Thus, clinicians who focus solely on risks and vulnerabilities of these children may fail to recognise the complexity of their experiences and may not respond appropriately to meet their needs.

Participants with a focus on the needs of children described becoming angry and judgmental towards parents for not adequately protecting and caring for their children. Similarly, Munroe [52] suggested that child protection clinicians may readily assign blame to parents. She argued that this perspective offers a straightforward explanation of the situation and a clear path of action to correct the behaviour of the parent. Likewise, one of the participants in this study, Kurt, believed it was necessary to adopt a non-supportive, authoritarian approach with parents to advocate for their children. He reported that this approach led to parents feeling judged and was detrimental to his rapport with them. This is understandable, given that parents who experience mental illness may already be worried about services judging or placing restrictions on their parenting or removing their children [26, 53] Furthermore, such an approach negates the significant social and economic disadvantage many parents with a mental illness experience and adds further to their disempowerment [53].

Several participants attempted to maintain a dual focus on parents and their children concurrently, despite some becoming preoccupied by the needs of children at times. Previous literature argues that a dual perspective is necessary to effectively work with parental mental illness, but also asserts that tensions exist between these two perspectives [34, 35]. Findings from this study affirm that such tensions were experienced by participants who adopted a dual focus. In contrast to participants with a singular focus on parents or children participants, those with a dual focus felt the pressure of meeting the needs of parents *and* the children. They worried that actions to support one party may negatively impact on the other. Therefore, the process of maintaining a dual focus involved actively and consciously juggling and balancing of the perspectives of parents and their children.

The tensions of maintaining a dual focus were eased when participants directed their efforts toward empowering parents to care for their children. In contrast to the assumption that the needs of children are unavoidably in conflict with the needs of parents [34, 35], a practice framework of parental empowerment allowed participants to unify the goals of supporting parents as well as meeting the needs of their children. An emphasis on parental empowerment also encouraged clinicians to recognise the strengths of parents and their children and eased feelings of sympathy and responsibility towards them. Thus, a model of parental empowerment is likely to assist clinicians to maintain a dual focus as well as relieve anxieties that are associated with working with parents [26].

Parental empowerment is a central goal of Let’s Talk about Children, a short intervention for working with parental mental illness [21]. During the Let’s Talk about Children intervention, clinicians encourage and support parents to identify and create positive changes within their family to strengthen relationships and build the resiliency of their children. Completion of online training in the intervention influenced clinicians’ attitudes relating to their work with parents, along with increased skill and knowledge [54]. Specifically, some clinicians realised, for the first time that positive family change could be achieved through parental empowerment. The promotion of this intervention, and the underlying principle of parent empowerment which promotes the view that parents may be the conduit of change back in their families, would support clinicians to manage a dual perspective of parents and children.

A recent study identified characteristics of clinicians associated with family-focused practices [55]. The strongest predictor of family-focused practices was prior training. Experienced, female clinicians practicing in rural locations were also more likely to engage in family-focused practices. The findings corroborate the importance of training which may be especially useful for clinicians who are inexperienced, male and/or working in populated locations. Moreover, the findings of the present study suggest that clinicians who are parents themselves may experience particularly strong emotional reactions towards parents and children. Further research is needed to determine the specific support that clinicians who are parents may need to manage feelings toward parents and children.

On the basis of this study and previous research, a number of recommendations can be made. Services need to support clinicians to employ a dual perspective when working with parents through strengths-based frameworks that emphasise parental empowerment. While policy is important, organisations and managers must also support clinicians by providing them with adequate time, resourcing and a workplace culture that affirms this complex and challenging work. Ongoing dialogue and support for working with issues related to parenting and child wellbeing may be provided by including this as a regular agenda item at regular meetings such as supervision and case reviews. It may also be beneficial to provide clinicians with a forum to discuss issues relating to parents and their children. Opportunities for consultation with specialists in the area of parental mental illness may also be of use. Additionally, clinicians should be trained in practice frameworks, such as the Let’s Talk about Children intervention, that promote concurrent support of parents and children through parental empowerment.

The voluntary sampling method utilised in this study is a limitation which may have attracted participants with a strong interest working with consumers who are parents. Although a range of professions were represented, the sample is skewed heavily towards psychologists and social workers. The sample size of 11 participants was appropriate for the contextualised, in-depth examination of their perspectives and experiences [56], but findings may not be generalisable. It is likely that clinicians may perceive and react to parents and children in additional ways to those identified in this study and that other factors may influence the focus of their work. Therefore, qualitative or survey-based methods with larger sample sizes are required to generalise and extend on these findings across the adult mental health workforce. The results of IPA are unavoidably influenced by the researchers’ preconceptions and biases [56]. All researchers of this study were advocates for the support of children and parents when parental mental illness is present. The influence of such biases were identified and moderated through the separate analysis of a subset of transcripts by the second author and conversations between all authors during all stages of the study.

Further research could investigate a range of areas related to clinicians’ perspectives of parents with a mental illness and their children. It would be beneficial to examine if/how clinicians’ perspectives are influenced by contextual factors such as the severity of the parental mental illness, the presence and supportiveness of the other parent or the age of children. Research could also examine the relationship between clinicians’ self-reported perceptions and actual practice. It is also crucial to develop and evaluate resources to support clinicians to maintain balanced perspectives of parents and children.

## Conclusions

The findings of this study indicate that a dual focus on parents and children may be difficult for mental health clinicians to maintain due to sympathetic feelings towards parents, children or both. Such feelings may lead clinicians to believe that the needs of parents conflict with the needs of children. Consequently, clinicians may feel torn between the two parties or compelled to take sides. Maintaining a balanced perspective of parents and children was supported by adopting a strengths-based framework of parental empowerment. It is vital that clinicians are supported by adult mental health services to employ such a framework and maintain a balanced perspective of parents and their children.

## Abbreviations

IPA: Interpretative Phenomenological Analysis
